# An assessment of non-volant terrestrial vertebrates response to wind farms—a study of small mammals

**DOI:** 10.1007/s10661-016-5095-8

**Published:** 2016-01-27

**Authors:** Rafał Łopucki, Iwona Mróz

**Affiliations:** Center for Interdisciplinary Research, The John Paul II Catholic University of Lublin, Konstantynów 1F, 20-708 Lublin, Poland; The John Paul II Catholic University of Lublin, Racławickie Avenue 14, 20-950 Lublin, Poland

**Keywords:** Wind farm, Environmental impact, Terrestrial animals, Small mammals, Rodents, Shrews

## Abstract

The majority of studies on the effects of wind energy development on wildlife have been focused on birds and bats, whereas knowledge of the response of terrestrial, non-flying vertebrates is very scarce. In this paper, the impact of three functioning wind farms on terrestrial small mammal communities (rodents and shrews) and the population parameters of the most abundant species were studied. The study was carried out in southeastern Poland within the foothills of the Outer Western Carpathians. Small mammals were captured at 12 sites around wind turbines and at 12 control sites. In total, from 1200 trap-days, 885 individuals of 14 studied mammal species were captured. There was no difference in the characteristics of communities of small mammals near wind turbines and within control sites; i.e. these types of sites were inhabited by a similar number of species of similar abundance, similar species composition, species diversity (*H*′ index) and species evenness (*J*′) (Pielou’s index). For the two species with the highest proportion in the communities (*Apodemus agrarius* and *Microtus arvalis*), the parameters of their populations (mean body mass, sex ratio, the proportion of adult individuals and the proportion of reproductive female) were analysed. In both species, none of the analysed parameters differed significantly between sites in the vicinity of turbines and control sites. For future studies on the impact of wind turbines on small terrestrial mammals in different geographical areas and different species communities, we recommend the method of paired ‘turbine-control sites’ as appropriate for animal species with pronounced fluctuations in population numbers.

## Introduction

Wind power is becoming increasingly important as a power supply in a growing number of countries. At the beginning of the twenty-first century, the global wind power capacity was 17 GW, and in 2013, it increased to 318 GW (REN21 2014). The promotion of this renewable source for electricity production is a priority in the energy policy of many countries (Karydis 2013; Mann and Teilmann 2013). Globally, the wind power capacity by the end of 2013 was sufficient to meet an estimated 2.9 % of total electricity consumption. In the European Union (EU), the operating capacity was able to cover nearly 8 % of total electricity consumption (in 2013), and several EU countries met higher shares of their energy demand with wind power, e.g. up to 20.9 % in Spain and 33.2 % in Denmark (REN21 2014).

However, the expansion of wind power has various environmental impacts (Carrete et al. 2012). Most studies about the effects of wind energy development and operation on wildlife have focused on flying species, specifically on avian and bat activities, habitat use and mortality (Baerwald et al. 2008; Drewitt and Langston 2006; Kunz et al. 2007; Pearce-Higgins et al. 2012), whereas very little has been published on the effects of wind energy on terrestrial, non-volant wildlife (de Lucas et al. 2005; Lovich and Ennen 2013; Santos et al. 2010). Lovich and Ennen (2013) concluded that more empirical data are currently needed to fully assess the impact of wind farms on non-volant wildlife. Although knowledge of this field is generally sparse, some research has shown that terrestrial animals can be affected by wind power development in various ways. The potential and known effects of wind farm construction and operation on terrestrial, non-flying species include the following: an increase in direct mortality; environmental impacts of destruction and modification of the habitat, including the impacts of roads, habitat fragmentation and barriers to gene flow; noise effects, visual impact, vibration and shadow flicker effects; electromagnetic field generation; macro- and micro-climate change; predator attraction and an increase in fire risks (Lovich and Ennen 2013).

A review of the literature on wind farm impacts on terrestrial, non-volant wildlife has shown that most studies were conducted on large-mammal carnivores and ungulates (Helldin et al. 2012; Lovich and Ennen 2013). Helldin et al. (2012) concluded that wind farms affect large mammals mainly through an increase in human activity at the wind farm area during the construction and operational phases. During the construction phase, large animals may temporarily avoid wind farms, but when construction and human presence is removed, animals acclimate to wind energy infrastructure. Low-level impacts of wind farms on the home ranges, behaviour and nutritional ecology of large mammals were observed. Meanwhile, during the operational phase of a wind farm, human presence increases (development of the network of access roads to the turbines may cause increased access for recreation, forestry, agriculture, hunting and leisure traffic), which affects large mammals via significant habitat loss.

Knowledge of the reaction of other groups of terrestrial, non-flying vertebrates is very scarce. Lovich and Ennen (2013) give examples of various studies on the impact of wind farms on Agassiz’s desert tortoise, a federally and state-protected species in the USA (CA) (Lovich et al. 2011), whereas ground-dwelling animals, such as small mammals (orders Rodentia and Soricomorpha) have been the subject of very limited studies. Small mammals are very common animals and can live in almost any terrestrial ecosystem, usually in multi-species communities. The species richness of small mammals is very high (Rodentia includes over 2300 species, Soricomorpha over 400 species) and constitutes approximately 50 % of worldwide mammalian biodiversity (Wilson and Reeder 2011). It is also known that human impacts may affect small mammals at the community and population level (e.g. urbanization [Łopucki et al. 2013], roads [Rico et al. 2007], agriculture [Horváth and Herczeg 2013]), and some species are threatened with extinction (Smulders et al. 2003).

Taking the above facts into account raises the question of whether the development of wind farms influences this group of animals. Thus far, this issue has not been sufficiently resolved in a scientific way. For example, de Lucas et al. (2005) concluded that wind farms did not clearly affect small mammal populations. In their studies, small mammal populations showed high variations in numbers, and this natural population fluctuation made it difficult to detect differences before, during and after wind farm construction. Rabin et al. (2006) presented a more specific study and hypothesized that the noise generated by wind energy turbines affected the behaviour of California ground squirrels (*Spermophilus beecheyi*). The species is highly social, and individuals vocalize to alert other members of the colony when a predator is detected. The results demonstrated a statistically significant effect of noise on squirrels at the turbine site; they showed increased caution and elevated vigilance in comparison with squirrels far away from turbines.

The aim of this work was to present empirical data of the impact of three functioning wind farms on terrestrial small mammals, i.e. rodents and shrews. We tested the null hypothesis of no effect (negative or positive) of wind turbines on communities of small mammals (species richness, species diversity, species evenness, community composition and relative abundance) and population parameters (mean body mass, sex ratio, the proportion of adult individuals and the proportion of reproductive females).

We assumed that the impact of wind turbines on small mammals should be the most distinct (and consequently the easiest to observe on a community or population level) in short-term functioning farms, because in such a short time, small mammals have not yet developed adaptations to this novel anthropogenic element in the environment. We also assumed, on the basis of the results of de Lucas et al. (2005), that long-term study of small mammals, conducted with the gradient transect method, may not show clear results. For this reason we decided to conduct a short-term study but in multi-repetition of paired ‘turbine-control sites’. We studied sites with the highest potential impact of wind turbines, i.e. sites close to wind turbines, and we tested effects of wind turbines on various communities and population parameters of small mammals. We expected that the effects, if they are significant, should be observed for at least some of the analysed parameters.

## Study area

The study was carried out in southeastern Poland (central Europe) within the foothills of the Outer Western Carpathian Mountains at three wind farms situated in the vicinity of the following localities: Łęki Dukielskie (N 49° 36′ 52″, E 21° 40′ 54″), Rymanów (N 49° 36′ 19″, E 21° 50′ 37″) and Bukowsko (N 49° 30′ 31″, E 22° 5′ 17″). The farms were located at distances of 11 and 19 km from each other. All farms are novel elements in the landscape of this region because they have been in operation for no longer than 5 years.

The Łęki Dukielskie wind farm consists of five Repower MM92 wind turbines with the following parameters: a tower height of 100 m and a rotor diameter of 92.5 m. The capacity of a single turbine amounts to 2.05 MW. The farm is located at an altitude of 370–410 m above sea level. The turbines are located next to a forest complex of an area of ca. 14 km^2^ at a distance of 18–600 m from the periphery of the forest. From the south, the turbines adjoin agricultural areas.

The Rymanów wind farm consists of 13 Repower MM92 wind turbines with the following parameters: a tower height of 100 m and a rotor diameter of 92.5 m. The capacity of a single turbine amounts to 2.05 MW. The farm is located at an altitude of 310–330 m above sea level. The turbines are surrounded by agricultural areas with arable fields and meadows. There are also small groups of shrubs located along the access roads, bounds and ditches and on non-managed patches of land.

The Bukowsko wind farm consists of nine Repower MM92 wind turbines with the following parameters: a tower height of 100 m and a rotor diameter of 92.5 m. The capacity of a single turbine amounts to 2.05 MW. The farm is located at an altitude of 520–580 m above sea level. The turbines stand in two groups located on adjacent hills. The study was conducted in a group consisting of three turbines, where from the east, the turbines are adjacent to a forested of an area of ca. 3 km^2^ at a distance of 70–150 m from the forest periphery, whereas from the west, they are adjacent to open, agricultural areas.

### Trapping sites

The capture of small mammals was conducted at 12 sites around the wind turbines and 12 control sites. This gave 12 pairs of turbine-control sites (Table 1).

**Table 1:** Habitat characteristics of the studied pairs of ‘turbine-control’ sites and the number of individuals captured in the study sites per 100 trap-days

No. of pairs of turbine-control sites	Habitat description of the studied sites	Number of individuals caught within the turbine and control sites and the trapping effort (number of indiv./trap-days)	
		Turbine sites	Control sites
1	Herbs and grasses with single shrubs and a small share of weedy and ruderal vegetation surrounded by a forest and agricultural areas	34/100	44/100
2	The periphery of a forest ecotone with herbs, grasses and groups of shrubs surrounded by a forest and cultivated fields	34/100	20/100
3	Unmanaged land around turbine towers with weedy and ruderal vegetation surrounded by cultivated fields	17/100	15/100
4	Unmanaged land around turbine towers with weedy and ruderal vegetation surrounded by cultivated fields	48/100	49/100
5	Unmanaged land around turbine towers with weedy and ruderal vegetation surrounded by cultivated fields	35/100	29/100
6	Wet, drained and moved meadows surrounded by the same type of meadows	43/100	44/100
7	Wet, drained and moved meadows surrounded by the same type of meadows	24/100	29/100
8	Herbs and grasses with single shrubs and a small share of weedy and ruderal vegetation surrounded by a forest and agricultural areas	39/100	35/100
9	Herbs and grasses with single shrubs and a small share of weedy and ruderal vegetation surrounded by a forest and agricultural areas	57/100	33/100
10	The periphery of a forest ecotone with herbs, grasses and groups of shrubs surrounded by a forest and cultivated fields	40/100	37/100
11	The periphery of a forest ecotone with herbs, grasses and groups of shrubs surrounded by forest and cultivated fields	35/100	57/100
12	The periphery of a forest ecotone with herbs, grasses and groups of shrubs surrounded by a forest and cultivated fields	44/100	43/100

For this study, we selected turbines with surroundings (affected environment) that provided a suitable habitat for small mammals, i.e. with a relatively dense undergrowth of wild (non-cultivated) plants. For the surroundings (affected environment), we assumed an area within a radius of approximately 60 m from the turbine tower, i.e. an area of ca. 1 ha. Captures were conducted if a habitat suitable for small mammals constituted at least 70 % of the closest surrounding of a turbine.

Vegetation types around the turbines were divided into the following three groups according to stages of ecological succession: (1) weedy and ruderal vegetation, which occurred around three turbines located within the agriculture area (nos. 3–5; Table 1); (2) herbs and grasses with single shrubs and a small share of weedy and ruderal vegetation, which occurred around three turbines located within the agricultural area usually a larger distance from a forest (nos. 1, 8 and 9; Table 1); and (3) herbs and grasses with numerous shrubs (hawthorn, willow, rose, dogwood, blackthorn), which occurred around four turbines located in an ecotone at the periphery of the forest (nos. 2 and 10–12; Table 1). Moreover, two turbines (nos. 6 and 7; Table 1) were located among wet mown meadows with *Sanguisorba officinalis* and *Cirsium canum.*

The study was conducted around two turbines in the Łęki Dukielskie wind farm (nos. 1 and 2; Table 1), around seven turbines in the Rymanów wind farm (nos. 3–9; Table 1) and around three turbines in the Bukowsko wind farm (nos. 10–12; Table 1). For each wind turbine site, the control site was chosen in such a way that the type of vegetation and its shared and occupied area, topography, altitude and the nature of the surroundings (type of ecosystems) were as similar as possible to those around the wind turbines. The control sites were located at a distance of 1.5–5 km from the corresponding turbine sites.

### Trapping scheme

Small mammals were captured in two types of traps: wooden box live traps (88 × 80 × 200 mm) and multiple-capture live traps with a metal mesh (Ugglan traps) (240 × 60 × 90 mm). All of the traps were provided with food bait. In each site, the traps were set along a transect consisting of 25 traps (15 wooden box live traps and 10 Ugglan traps) spaced at 15-m intervals. Within the wind turbine sites, the traps were set in a radius of 60 m from the turbine tower. Within the control sites, the traps were set in a similar area (i.e. approximately 1 ha.). In the wind turbine sites and linked control sites, captures were carried out in the same time; moreover, the number of traps, the time of day and duration that the traps were open were the same. The traps were monitored twice a day: in the morning and in the evening. One trapping session lasted for 4 days. The capture effort of each site was 100 trap-days. Captured animals were described in terms of species, sex, reproductive activity and body mass (±1 g). The reproductive activity of males was determined on the basis of visible testes. The reproductive activity of females was determined on the basis of visible pregnancy or lactation. The sex of Soricomorpha species was not determined due to lack of sexual dimorphism. Newly captured individuals were marked by fur clipping. After handling, all of the individuals were released at the site of capture. Trapping sessions were carried out in the breeding season of small mammals, i.e. in the summer (from July to September) of 2014.

### Data analysis

Communities of small mammals within the wind turbine and control sites were analysed on the basis of (1) species richness, defined as the number of species recorded at each site, and were compared between wind turbine and control sites using a non-parametric Mann-Whitney U test; (2) species diversity, calculated using the Shannon-Wiener index (*H*′) for each wind turbine or control site using a natural logarithm and compared between these sites using the Mann-Whitney *U* test; (3) species evenness presented by Pielou’s index (*J*′) and calculated for each wind turbine and control site, and compared between them using the Mann-Whitney *U* test; (4) community composition at the wind turbine and control sites, characterized by species dominance, calculated as the number of individuals of a given species divided by the total number of individuals of all species and expressed as a percentage; and (5) relative abundance of small mammal at each wind turbine and control site, determined as the number of individuals of particular species per 100 trap days and compared using the Mann-Whitney *U* test; relative abundance was compared for entire small mammal community as well as for rodents and *Soricomorpha* separately.

We only analysed the population parameters for the most numerous species with the highest contribution in communities. We analysed the following parameters: (1) body mass (g), presented as a mean with minimum-maximum weight and compared between the wind turbine and control sites with Student’s *t* test; (2) the proportion of adult individuals of a given species with a body mass above 20 g, compared between wind turbine and control sites with a chi-square test; (3) sex ratio, expressed as the proportion of females and males in a population of given species and then compared between wind turbine and control sites with a chi-square test; and (4) the proportion of reproductive females (visible pregnancy or lactation) in a population of a given species and compared between wind turbine and control sites with a chi-square test.

Statistical tests were performed using STATISTICA version 10.0 (StatSoft, Inc., Tulsa, OK, USA).

## Results

### Community composition

**Fig. 1:**
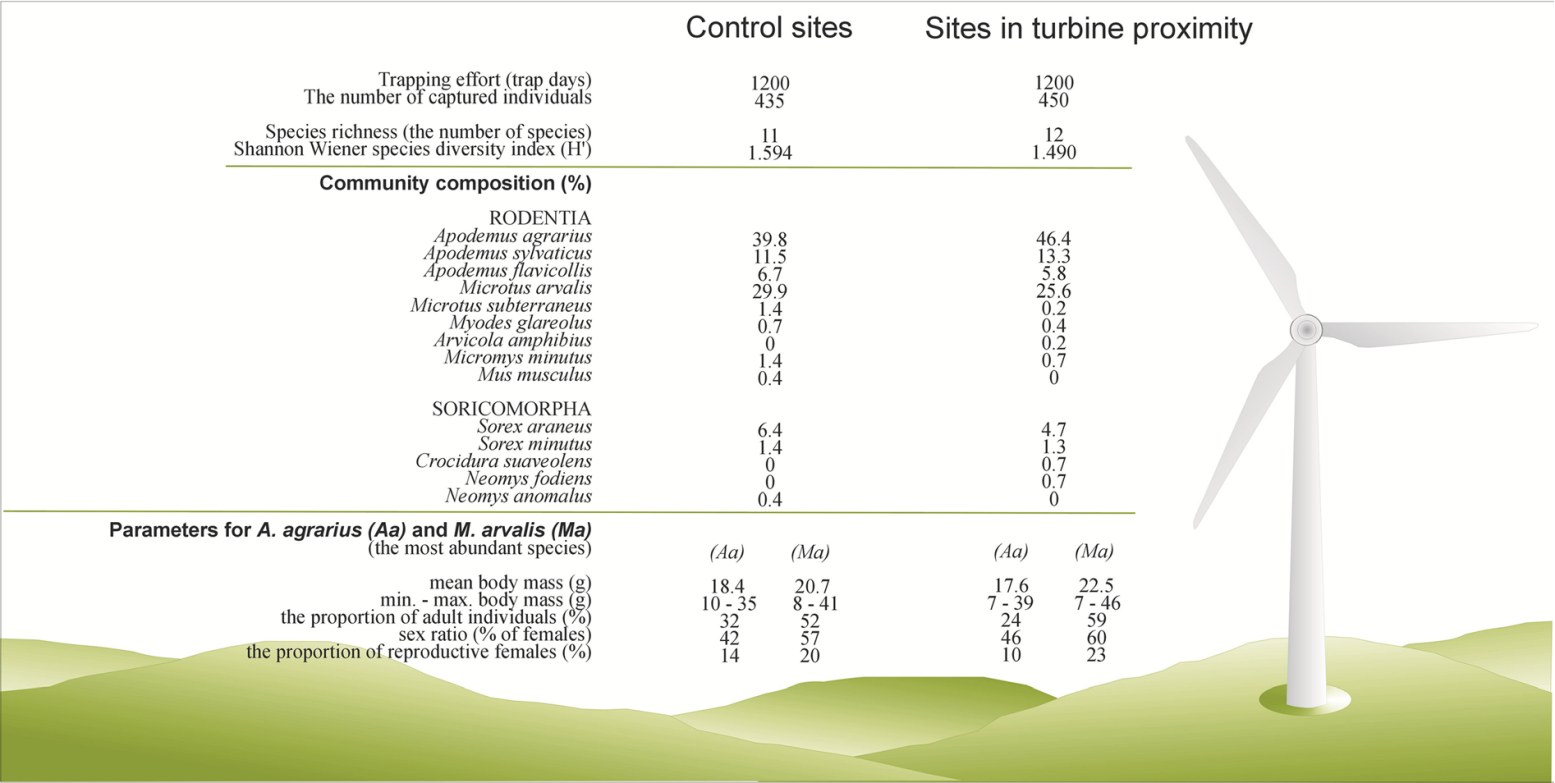
Species composition and proportion of small mammal species (%), species richness, species diversity (*H*′ index) and population parameters of the most abundant species (*Apodemus agrarius* and *Microtus arvalis*) within sites in turbine vicinity and control sites

In total, from 2400 trap-days, 885 individuals of 14 species of small mammals were caught, including 816 individuals of 9 rodent species and 69 individuals of 5 species belonging to the family Soricidae (order Soricomorpha) (Fig. 1, Table 1).

**Fig. 2:**
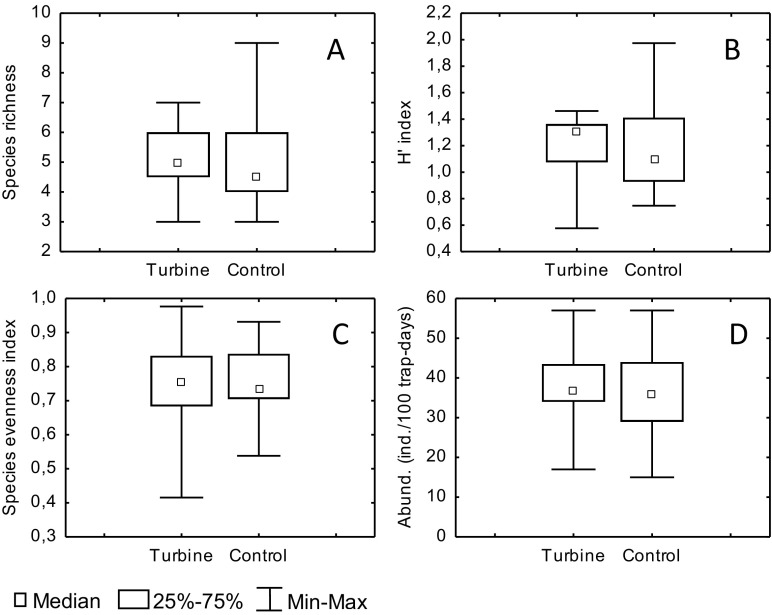
Comparison of indices: species richness (**a**), species diversity (*H*′ index) (**b**), species evenness (*J*′) (Pielou’s index) (**c**) and relative abundance (**d**) of small mammals between turbine and control sites. Significant differences were not found in any case (*p* > 0.05, Mann-Whitney *U* test)

The species richness of small mammals found at the wind turbine and control sites was 12 and 11 species respectively. The species richness medians for particular wind turbine and control sites were 5 and 4.5 respectively, and these did not differ significantly (*Z* = 0.38, *p* = 0.7) (Fig. 2a). The index of species diversity (*H*′) for communities of small mammals around the turbine and control sites (with the medians *H*′ = 1.313 and *H*′ = 1.096 respectively) also did not differ significantly (*Z* = 0.46, *p* = 0.6) (Fig. 2b), nor did species evenness (*Z* = 0.11, *p* = 0.9). The median species evenness indices at the turbine and control sites amounted to *J*′ = 0.755 and *J*′ = 0.733 respectively (Fig. 2c).

The species composition was similar within both types of sites. Around wind turbines as well as in control sites, two species predominated: *Apodemus agrarius*, with a proportion in the communities amounting to 46 and 40 % respectively, and *Microtus arvalis*, with a proportion amounting to 26 and 30 % respectively. The proportion of *A. sylvaticus*, *A. flavicollis* and *Sorex araneus* in these communities was much lower and ranged between 5 and 13 % without significant differences between the turbine and control sites. Other species constituted a small percentage of communities, i.e. below 1.4 % (Fig. 1). Values of the species evenness index (Fig. 2c) indicate that in both communities (at the turbine and control sites) exhibit a lack of explicit domination among species.

The relative abundance of all small mammals did not differ significantly between the wind turbine (median 37) and control sites (median 36) (*Z* = 0.23, *p* = 0.8) (Fig. 2d). The relative abundance of rodents also did not differ significantly between the turbine (median 33) and control sites (median 33) (*Z* = 0.26, *p* = 0.8), nor did the relative abundance of shrews (Soricidae) (*Z* = −0.38, *p* = 0.7), with medians of 3 and 3 respectively.

### Population parameters

Population parameters were analysed for the two species with the highest relative abundance in the communities, *A. agrarius* and *M. arvalis* (Fig. 1).

In *A. agrarius*, the mean body mass of individuals captured around the wind turbines (18.4 g) and at control sites (17.6 g) did not differ significantly (*t* = −1.4, df = 380, *p* = 0.17) (Fig. 1). The proportion of adult individuals, i.e. with a body mass above 20 g, was similar within both types of sites and also did not differ significantly (*χ*^2^ = 3.4, df = 1, *p* = 0.07). The sex ratio did not show differences between the wind turbine and control sites (*χ*^2^ = 0.9, df = 1, *p* = 0.35), nor did the proportion of reproductive females (*χ*^2^ = 0.5, df = 1, *p* = 0.48) (Fig. 1).

Additionally, in *M. arvalis*, none of the analysed parameters differed significantly between the turbine and control sites: mean body mass (*t* = 1.7, df = 243, *p* = 0.09) (20.7 and 22.5 g respectively), proportion of adult individuals (*χ*^2^ = 1.4, df = 1, *p* = 0.2), sex ratio (*χ*^2^ = 0.2, df = 1, *p* = 0.62) and proportion of reproductive females (*χ*^2^ = 0.2, df = 1, *p* = 0.67) (Fig 1).

## Discussion

The potential negative effects of wind farm operations on terrestrial small mammals may include noise, vibration, electromagnetic and visual effects as well as the impact of access roads by increasing direct mortality or habitat fragmentation (Lovich and Ennen 2013). Additionally, a positive impact can also be considered (de Lucas et al. 2005), because wind farm may reduce the occurrence of birds of prey (Pearce-Higgins et al. 2009), and in consequence small mammals living near the turbines may be under less pressure from avian predators. Such effects (negative or positive), if they are significant, should be observed at the community (e.g. lower species richness [Santos et al. 2010]) or population level. For example, more sensitive small mammal species may avoid the proximity of wind turbines, and their contribution in the small mammal community should be lower than within control sites. This phenomenon is observed in both rodents and shrews in urban areas with high and diverse human impacts (Łopucki and Kiersztyn 2015; Łopucki and Kitowski 2014). Second, high noise, movement of wind turbine blades and vibration may cause the sites near the turbines to become presumably less suitable as potential habitats (the sound can disrupt animal vocal communication or impair the animals’ ability to hear approaching predators (Helldin et al. 2012)), so the abundance of those species would be lower there. Furthermore, the suboptimal characteristics of these habitats may result in differences in the level of reproduction or social structures of animal populations occurring there. Theoretically, according to the ‘habitat selection model’, the optimal habitats are occupied by adult breeding individuals of high social position, while the subordinate individuals (younger, often reproductively inactive) are forced towards lower-quality habitats (Halama and Dueser 1994).

However, our study shows that at the community level, there are no significant differences between small mammals occurring at wind farms and their counterparts from the control sites. The studied sites were inhabited by a similar number of species of similar abundance, similar species composition species diversity (*H*′ index) and species evenness (Pielou’s index). This means that the null hypothesis considered in the work was confirmed – there are no differences in the studied communities; thus, there is no evidence of the impact (negative or positive) of operating wind farms upon small mammals based on the variables and species we studied during the short-term presence and operation of turbines.

Regarding the population parameters (none of the analysed parameters differed significantly between the turbine and control sites), more careful conclusions should be drawn. First, the analysis was based only on simple indicators assessed visually without measuring chemical characteristics, e.g. through stress parameters or genetic tests. It cannot be excluded, however, that with a more detailed analysis, such differences in behavioural and physiological responses could be observed (Gauffre et al. 2008; Mikołajczak et al. 2013; Rabin et al. 2006; Wang et al. 2011). Second, the analysis was conducted only for two species because the results for other species could be insignificant due to their low number. The species analysed, *A. agrarius* and *M. arvalis*, differ ecologically and belong to different taxonomic groups. *A. agrarius* belongs to the Muridae family and is a species occupying a broad habitat and food niche, and it usually does not form family social groups (Gliwicz and Kryštufek 1999). On the other hand, *M. arvalis* belongs to the Cricetidae family, and it prefers mainly open habitats, feeds on the green parts of grasses and herbaceous plants and lives in family groups (Zima 1999). Nonetheless, despite explicit differences between these species, in our work, we obtained similar results – none of the analysed parameters differed significantly between turbine and control sites.

It should be also noted, that our study was conducted in a certain range of small mammals’ abundance, which correspond to middle or middle-high densities of small mammals (Pupila and Bergmanis 2006). Metrically, this relative abundance was about 37 (median value) or 37.5 (mean value) individuals captured per 100 trap-days at turbine sites and 36 (median) or 36.25 (mean) at control sites. This abundance can be also expressed (recalculated) as the number of individuals per hectare. For example using the algorithm of Jareño et al. (2014), it can be estimated that abundance of common vole *M. arvalis* at our study sites range from 4.5 to 13.6 individuals/ha. The question is whether in the case of other densities the effect of wind turbines on small mammals may be different. We hypothesize that it is possible in the case of lower densities of small mammals. It is known, that habitats are filled in order of quality (Székely et al. 2010); first, optimal habitats become filled and then the suboptimal habitats until there are no suitable habitats available for occupancy. In low densities of small mammals, when optimal habitats are not filled, the habitats near turbines cannot be occupied due to their suboptimal character. In the case of high densities of small mammals, we expected similar effect, as was described in our study.

The method of paired turbine-control sites applied in this work, assuming the comparison of the parameters between the sites with the strongest impact of a wind turbine and well-chosen control sites, seems to be the most simple and useful method for analysing the impact of wind power on small mammals. Used in the work of de Lucas et al. (2005), the method of line transects covering a wind farm area can yield ambiguous results due to running trap-lines through a variety of habitats (or micro-habitats) and a synergistic effect of adjacent wind turbines. Similarly, the study design, known as the before-after control-impact (BACI) (Helldin et al. 2012; Kuvlesky et al. 2007), can rarely be used in studies of small mammals. de Lucas et al. (2005) noted that the natural annual fluctuations in the abundance of small mammals make it difficult to detect differences before, during and after wind farm construction. Small mammals are, in fact, a group of animals with more or less predictable fluctuation patterns, and the differences in abundance between years can amount to several thousand percent (Krebs 2013). Carrying out a single study using the BACI method, a natural number of fluctuations in rodent populations may affect the results and lead to false conclusions. Another reason that the BACI method is not appropriate in studies on small mammals is the lack of widely available data on the population parameters of these animals before wind farm construction. The deficiency of these data results from the fact that the impact of the construction of a wind farm on small mammals is rarely raised in the process of environmental impact assessments (EIAs). The lack of reporting by EIAs on the occurrence, species composition and abundance of small mammals before wind farm construction results in a deficiency of reliable data for comparison during wind farm operation. The only exception may be the monitoring of rare and protected species of rodents. In Poland, examples of such species are the speckled ground squirrel *S. suslicus* and European hamster *Cricetus cricetus*, for which monitoring is usually performed before wind farm construction and recommended during and/or after its operation (Łopucki and Dejneka 2013). Currently, there is a lack of long-term studies summarizing the results of such studies.

Summarizing, although wind farms could potentially affect small mammals in various ways, our study showed no significant effect of operating wind turbines upon the communities and population parameters of rodents or shrews. Because the work was conducted at three wind farms located in the same geographical region, further similar studies (in different geographical areas and in different communities of mammals) are needed to explicitly confirm our observations. Moreover, studies carried out in different densities of small mammals and different ages (operating time) of wind farm are needed to fully recognize the effect of wind power on this group of terrestrial mammals. We consider that the study method that we used (paired near-turbine study sites and well-matched control sites) may be the best one for determining the impact of wind turbines on small terrestrial mammals characterized by large fluctuations in population numbers.
